# Case Report: Marked elevation of serum procalcitonin before radiographic confirmation of recurrence in stage IVB pulmonary large cell neuroendocrine carcinoma

**DOI:** 10.3389/fmed.2026.1904660

**Published:** 2026-09-14

**Authors:** Yian Wang, Jiameng Lu, Xiaoqing Ji, Ran Zhang, Shuran Yang, Zhenlei Zhang, Degan Lu

**Affiliations:** 1 Department of Respiratory, The First Affiliated Hospital of Shandong First Medical University & Shandong Provincial Qianfoshan Hospital, Shandong Institute of Respiratory Diseases, Shandong Institute of Anesthesia and Respiratory Critical Medicine, Jinan, Shandong, China; 2 Faculty of Medicine, Macau University of Science and Technology, Taipa, Macau Special Administrative Region, China; 3 Department of Nursing, The First Affiliated Hospital of Shandong First Medical University & Shandong Provincial Qianfoshan Hospital, Jinan, Shandong, China

**Keywords:** early detection, indicator, large cell neuroendocrine carcinoma, procalcitonin, tumor recurrence

## Abstract

Serum procalcitonin (PCT) is conventionally used as a biomarker for bacterial infection, however, emerging evidence suggests that PCT may also be elevated in patients with lung cancer in the absence of infection. We report a patient with stage IVB pulmonary large cell neuroendocrine carcinoma (LCNEC) who experienced tumor recurrence after achieving partial response(PR) with multimodal therapy. During remission, serum PCT remained relatively stable at a baseline level of 1.62 ng/mL (normal range: 0–0.05 ng/mL). Subsequently, PCT increased markedly to 61.57 ng/mL despite the absence of definitive radiographic progression and rose further to 93.14 ng/mL when tumor recurrence was ultimately confirmed. Notably, marked elevation in PCT preceded radiographic confirmation of recurrence by more than one month, with no clinical or microbiological evidence of concurrent infection. This biochemical lead time suggests that serial PCT monitoring may have potential value as an adjunctive biomarker for the early detection of recurrence in advanced LCNEC, although further studies are required to establish its clinical utility.

## Introduction

Procalcitonin (PCT) is a 116-amino acid polypeptide that is physiologically synthesized by the C cells of the thyroid gland as a precursor to calcitonin (Cal) (1, 2). It is widely used as a biomarker to support the diagnosis and clinical assessment of bacterial infections, particularly severe systemic infections (3). However, elevated serum PCT levels have also been reported in patients with various malignancies in the absence of overt infection (4). Increasing evidence further suggests an association between PCT elevation and lung cancer, particularly tumors with neuroendocrine differentiation (5).

Large cell neuroendocrine carcinoma (LCNEC) is a rare, high-grade pulmonary neuroendocrine tumor, accounting for approximately 3% of all lung carcinomas (6). Although PCT expression has been detected in a subset of LCNEC tumors (7), the clinical significance of longitudinal changes in serum PCT for monitoring treatment response and detecting disease recurrence remains poorly defined. Here, we report a patient with stage IVB LCNEC who exhibited a marked elevation in serum PCT in the absence of clinical, microbiological, or radiographic evidence of infection. Notably, the increase in PCT preceded radiographic confirmation of tumor recurrence, suggesting that serial PCT monitoring may warrant further investigation as a potential adjunctive biomarker for post-treatment surveillance in LCNEC.

## Case presentation

A 47-year-old woman was admitted to our hospital on April 1, 2025 because of worsening paroxysmal cough with scanty white mucoid sputum and exertional dyspnea. She had no smoking history and reported only occasional alcohol consumption. In November 2023, she had been diagnosed with LCNEC of the right lower lobe (Figures 1A,B), clinically staged as cT1N3M1c (IVB), with metastases to the liver, bone, and multiple lymph node stations. Histopathological examination of a computed tomography (CT)-guided needle biopsy pathology revealed a high-grade neuroendocrine carcinoma with morphological features consistent with LCNEC (Figure 1C). Immunohistochemistry staining showed tumor-cell positivity for cytokeratin (CK), synaptophysin (Syn), CD56, insulinoma-associated 1 (INSM1), chromogranin A (CgA), and thyroid transcription factor 1 (TTF-1), with a Ki-67 proliferation index of approximately 60%. Collectively, the morphological and immunohistochemical findings support the diagnosis of pulmonary LCNEC. At the time of initial diagnosis, the serum levels of PCT and pro-gastrin-releasing peptide (Pro-GRP) were 1.49 ng/mL (normal range: 0–0.05 ng/mL) and 210.00 pg/mL (normal range: <65.00 pg/mL), respectively.

**Figure 1:**
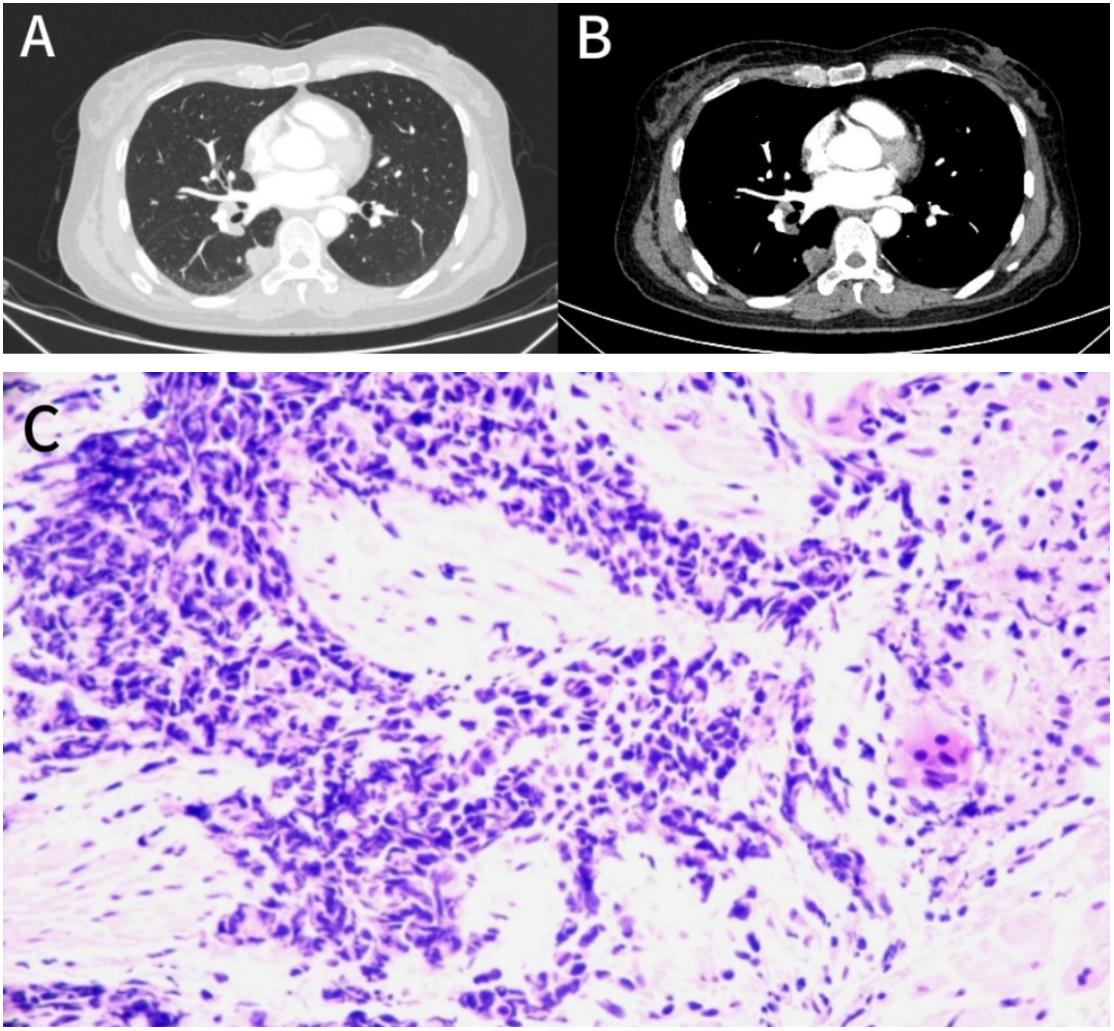
Chest computed tomography and pathological biopsy. **(A)** Lung window revealed a mass lesion in the right lower lobe. **(B)** Mediastinal window showed the corresponding soft-tissue density mass with associated mediastinal lymph node metastasis. **(C)**The cells are large with a low nuclear-to-cytoplasmic ratio, relatively abundant cytoplasm, and a sheet-like distribution (hematoxylin-eosin, original magnification  ×  400).

The patient subsequently received multimodal treatment, including hepatic ablation, six cycles of platinum-based combination chemotherapy, followed by maintenance therapy with a paclitaxel-based regimen in combination with anlotinib, an antiangiogenic agent. Following treatment, the overall tumor burden was substantially reduced. Follow-up CT examinations in September 2024 demonstrated a partial response (PR) with sustained disease control (Figures 2A,B), while serum PCT remained relatively stable at 1.62 ng/mL and Pro-GRP was 50. 30 pg/mL. In October 2024, CT showed a slight increase in tumor size (Figures 2C,D), accompanied by a mild elevation of serum PCT to 3.22 ng/mL, whereas the Pro-GRP level was 69.80 pg/mL. Subsequent assessment in December 2024 showed stable disease (SD) (Figures 2E,F), with no significant change in PCT level.

**Figure 2:**
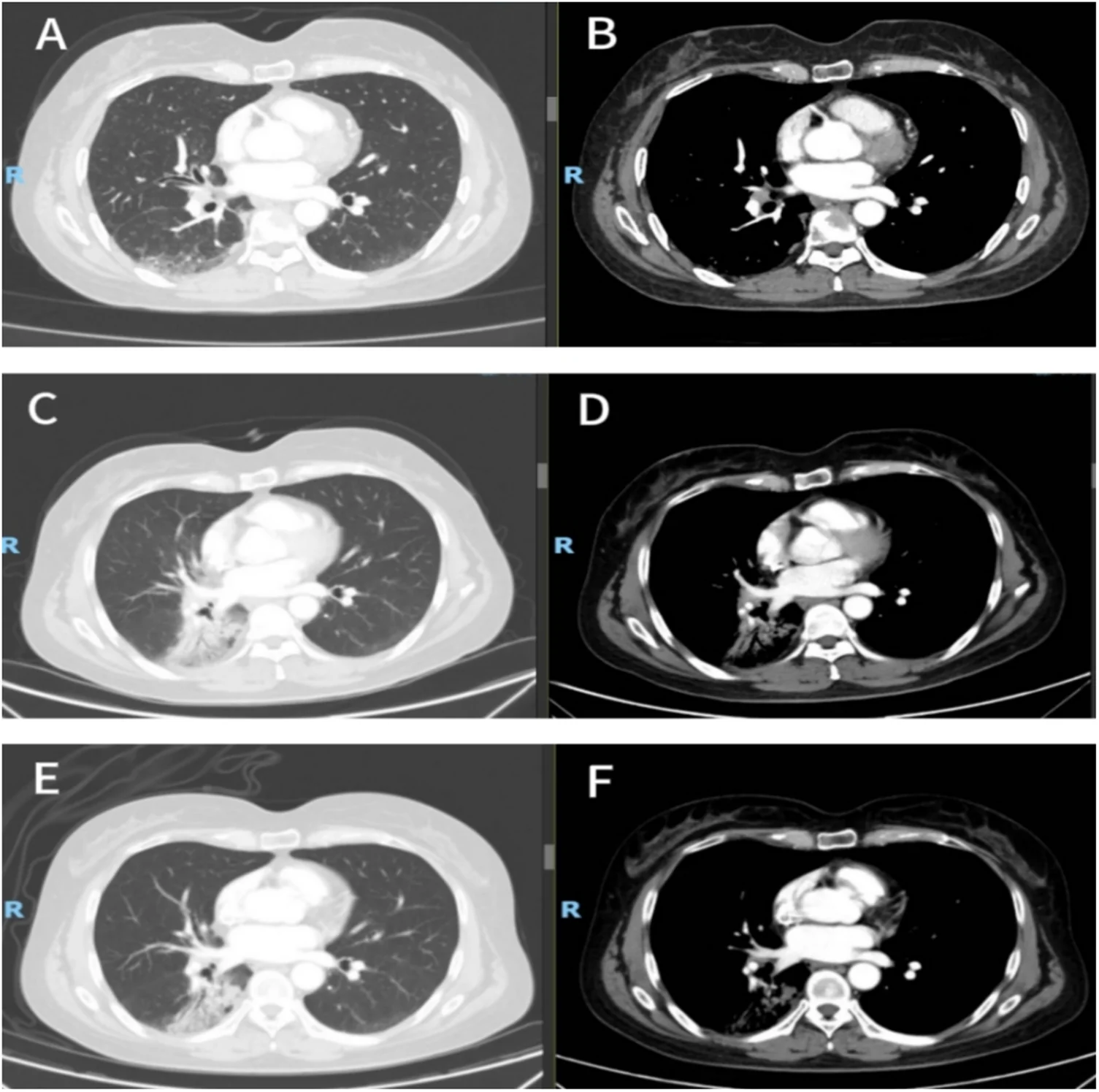
Serial chest CT images documenting treatment response during 2024. **(A,B)** Lung and mediastinal window views in September 2024 showed initial reduction in tumor burden following multimodal therapy. **(C,D)** Follow-up imaging in October 2024 demonstrated slight progression of the primary lesion and nodal disease. **(E,F)** Restaging CT in December 2024 confirmed stable disease.

In February 2025, follow-up CT showed no definite radiographic evidence of progression (Figures 3A,B). In contrast, serum PCT increased markedly to 61.57 ng/mL, representing a substantial rise from the preceding relatively stable levels, while Pro-GRP showed a modest increase to 335.80 pg/mL. At that time, the patient was afebrile and hemodynamically stable, with a temperature of 36.4 °C, heart rate of 80 beats/min, respiratory rate of 16 breaths/min, and blood pressure of 135/77 mm Hg. A comprehensive evaluation for infectious and inflammatory causes of PCT elevation, including microbiological and immunological investigations, did not identify an alternative explanation for the marked PCT increase; the detailed results are summarized in Table 1.

**Figure 3:**
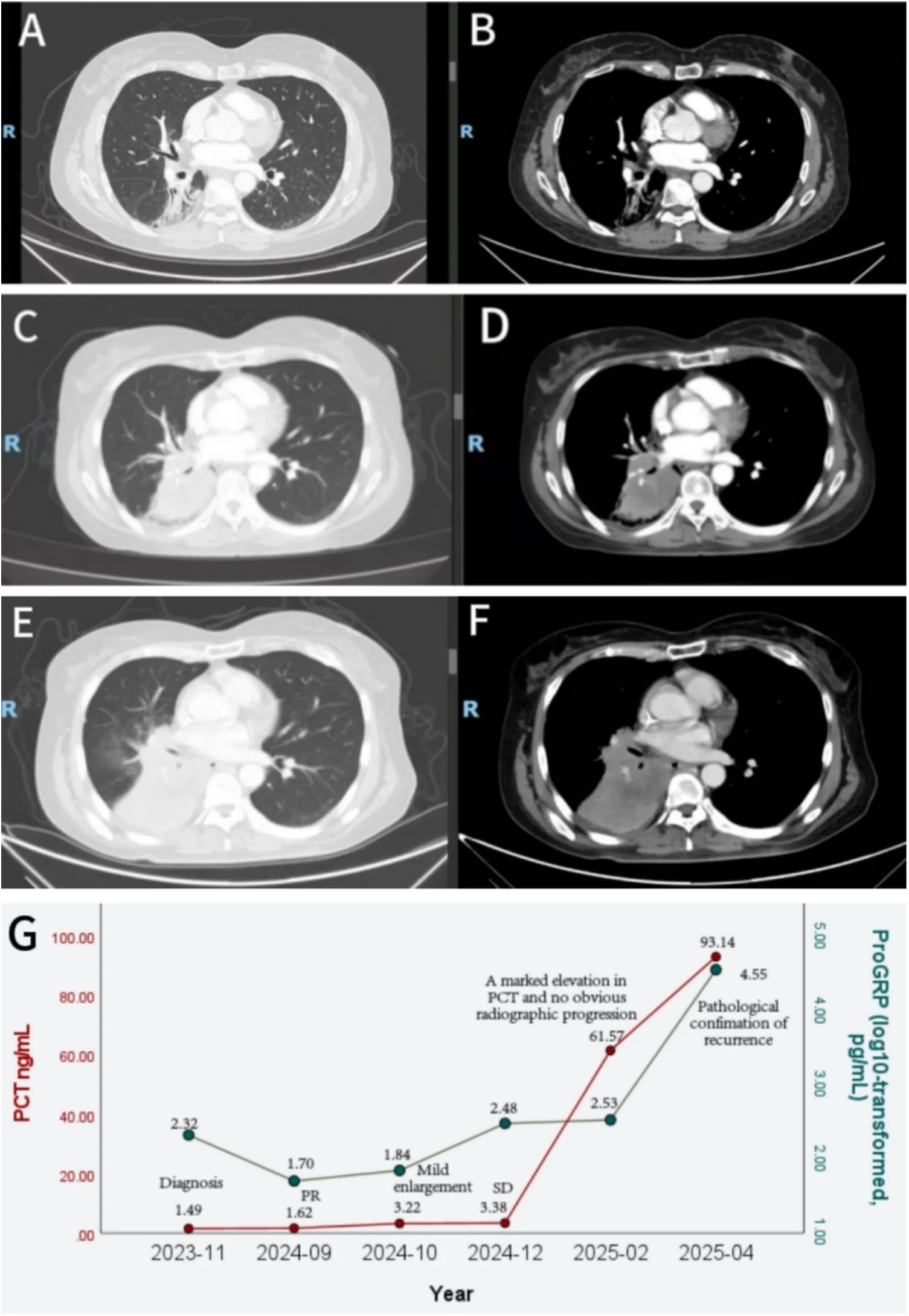
Chest computed tomography findings at tumor recurrence and serial changes in serum procalcitonin and pro-gastrin-releasing peptide levels correlated with key clinical events. **(A,B)** Lung and mediastinal window views in February 2025 showed no obvious radiographic progression. But serum PCT had markedly elevated to 61.57 ng/mL. **(C,D)** Lung and mediastinal window views in March 2025 revealed marked enlargement of the right lower lobe soft-tissue mass, measuring approximately 4.9 × 5.7 cm. The lesion showed progressive growth with involvement of the adjacent hilar and mediastinal structures. Enlarged lymph nodes were observed in the right hilum, mediastinum (stations 2R, 4R, 4L, 5, and 7), and left supraclavicular region. Serum PCT had further increased to 93.14 ng/mL. **(E,F)** Lung and mediastinal window views in May 2025 demonstrated further enlargement of the right lower lobe and mediastinal mass, with new pericardial involvement. **(G)** PCT remained low during remission and rose markedly to 61.57 ng/mL in February 2025, prior to definitive radiographic progression (March 31) and pathological confirmation of recurrence (April 2025). Pro-GRP values are presented as log_10_-transformed concentrations. Pro-GRP levels increased during disease progression.

**Table 1:** Microbiological, inflammatory, and immunological findings in February and April 2025.

Test	February 2025	April 2025	Reference/expected result	Unit
Bacterial examinations				
TB-DNA	Negative	Negative	Negative	—
Throat swab bacterial culture and identification	Viridans streptococci, *Neisseria sicca*	Viridans streptococci, *Neisseria sicca*		—
Sputum bacterial culture	Viridans streptococci, *Neisseria sicca*	Viridans streptococci, *Neisseria sicca*	—	—
BAL fluid bacterial culture and identification	Viridans streptococci; no *Haemophilus* growth	Viridans streptococci; no *Haemophilus* growth	—	—
Viral examinations				
CMV-DNA	Below detection limit	Below detection limit	<500	copies/mL
Fungal examinations				
G test	19.2		<151.5	pg/mL
GM test	0.20		<0.8	—
*Pneumocystis jirovecii* nucleic acid	Negative	Negative	Negative	—
Inflammatory marker				
ESR	**22**	**21**	0–20	mm/h
Immune status				
Total T-cell (CD3⁺) count	492	**469**	877–2,310	/*μ*L
CD4⁺ T-cell count	187	**180**	456–1,261	/μL
B-cell (CD19⁺) count	**13**	**11**	96–412	/μL
NK-cell (CD16⁺) count	**39**	**33**	110–780	/μL

BAL, bronchoalveolar lavage; TB-DNA, Mycobacterium tuberculosis DNA; CMV-DNA, Cytomegalovirus DNA; G test, (1→3)-β-D-glucan assay; GM test, Aspergillus galactomannan assay; ESR, erythrocyte sedimentation rate.

Beginning in early March 2025, the patient gradually developed recurrent respiratory symptoms, predominantly worsening dry cough and exertional dyspnea. Empirical antibiotic therapy initiated at an outpatient facility yielded no meaningful symptomatic improvement, and she was subsequently admitted to our hospital on April 1, 2025. On admission, her vital signs were stable, and she was afebrile, with a temperature of 36.2 °C. Chest CT performed on March 31, 2025 revealed a significant enlargement of the right lower lobe mass compared with prior imaging obtained during disease control, with the lesion measuring approximately 4.9 cm × 5.7 cm (Figures 3C,D). In addition, progressive lymphadenopathy was observed in the right hilum, mediastinum (stations 2R, 4R, 4L, 5, and 7), and the left supraclavicular region, findings highly suggestive of disease recurrence. Repeat serum PCT measurement on April 2, 2025 showed a further increase to 93.14 ng/mL. At that time, there remained no compelling clinical or microbiological evidence of active infection (Table 1). Meanwhile,Pro-GRP surged dramatically to 35567.00 pg/mL.Histopathological confirmation of recurrence was obtained by repeat bronchoscopic biopsy on April 2, 2025, which again demonstrated LCNEC with a morphological and immunohistochemical profile consistent with that of the original tumor. Follow-up chest CT on May 6, 2025 demonstrated further interval progression of the thoracic disease, including enlargement of the mediastinal mass and newly apparent pericardial involvement (Figures 3E,F).

Importantly, the initial marked increase in serum PCT to 61.57 ng/mL had already been documented in February 2025, when CT showed no definite radiographic evidence of progression. Thus, the substantial PCT elevation preceded radiographic confirmation of recurrence by more than one month.Serial changes in serum PCT in relation to the major clinical and radiographic events from initial diagnosis through disease recurrence are illustrated in Figure 3G. This temporal pattern raises the possibility that a marked rise in serum PCT may precede overt radiographic progression in LCNEC and supports further investigation of serial PCT measurements as a potential adjunctive biomarker for disease surveillance.

## Discussion

In the present case, a 47-year-old woman with stage IVB pulmonary LCNEC experienced disease recurrence after achieving substantial tumor reduction following multimodal therapy. During the period of disease control, serum PCT remained relatively stable at approximately 1.62 ng/mL. In February 2025, however, PCT increased abruptly to 61.57 ng/mL despite the absence of definite radiographic progression and subsequently rose further to 93.14 ng/mL when recurrence was confirmed histologically and radiographically Importantly, the patient remained afebrile, and extensive microbiological evaluation did not identify evidence of active infection.The marked increase in PCT preceded radiographic confirmation of recurrence by more than one month, raising the possibility that biochemical changes in circulating PCT may occur before overt morphological progression becomes detectable on conventional imaging. Previous studies have reported elevated PCT levels in patients with advanced solid tumors have suggested associations with tumor burden, disease progression and unfavorable prognosis. Stando and Chmielewski documented a patient with stage IV lung adenocarcinoma in whom PCT rose from 9.83 to 880.16 ng/mL in the absence of documented infection, in parallel with progressive disease and the development of new metastatic lesions (8). In small cell lung cancer (SCLC), Ichikawa et al. prospectively demonstrated that elevated pretreatment PCT was associated with poorer clinical outcomes (9). Pardo Cabello et al. further reported that serum PCT levels in metastatic SCLC increased with greater tumor burden and declined after effective chemoradiotherapy (10). Collectively, these observations suggest that circulating PCT may, in selected neuroendocrine or advanced thoracic malignancies, reflect changes in tumor-associated biological activity. However, most published studies have focused on baseline PCT levels, established tumor burden, or prognosis rather than on longitudinal PCT changes during post-treatment surveillance. The present case therefore adds a potentially informative temporal observation: serum PCT remained relatively stable during disease control and subsequently increased markedly before definite radiographic recurrence was identified. Although a single case cannot establish diagnostic sensitivity or specificity, this pattern supports further investigation of serial PCT measurement as a potential adjunctive biomarker for detecting early biochemical evidence of relapse in LCNEC. The biological mechanism underlying tumor-associated PCT elevation remains incompletely understood. In this patient, the marked PCT increase occurred in the absence of clinical or microbiological evidence of infection and was temporally associated with tumor recurrence, making a tumor-related source biologically plausible. LCNEC and SCLC share several clinicopathologic and molecular characteristics, including neuroendocrine differentiation and expression of neuroendocrine-associated gene (11). Itoga et al. reported immunohistochemical PCT expression in approximately 50% of examined LCNEC specimens and observed an association between serum PCT levels and disease status (7). These findings provide direct for the possibility that at least a subset of LCNECs may produce PCT or PCT-related peptides and that tumor-derived production could contribute to elevated circulating concentrations.

Additional mechanisms may also be involved. Systemic and tumor-associated inflammatory signaling may stimulate extrathyroidal PCT production. Pro-inflammatory cytokines, including tumor necrosis factor-α (TNF-α), interleukin-6 (IL-6), and interleukin-1 (IL-1), have been implicated in the regulation of PCT expression under inflammatory and stress-related conditions, while hypoxia and an inflammatory tumor microenvironment may further amplify these pathways (12). Nevertheless, the extent to which these mechanisms contribute specifically to PCT elevation in LCNEC remains uncertain. Hepatic involvement may represent another contributing factor, as markedly elevated PCT concentrations have been described in patients with advanced malignancies involving the liver (13). Given the presence of hepatic metastases in the present patient, a contribution from liver tumor burden or altered hepatic inflammatory signaling cannot be excluded. In addition, lymphopenia observed during disease progression may reflect systemic immune dysregulation associated with advanced malignancy; however, its mechanistic relationship with PCT elevation remains speculative and should not be inferred directly from this single case. From a clinical perspective, the possibility that serial PCT changes may precede radiographic recurrence is of potential interest because surveillance of advanced LCNEC currently relies predominantly on clinical assessment and serial imaging, often following principles extrapolated from SCLC owing to the rarity of LCNEC and the limited disease-specific evidence base (14). Conventional serum tumor markers, including carcinoembryonic antigen (CEA) and neuron-specific enolase (NSE) may also be used in selected patients, although their sensitivity and specificity for early recurrence remain imperfect (15). Pro-GRP has been investigated as a biomarker for disease status and treatment response in pulmonary neuroendocrine tumors, particularly SCLC, while its role in LCNEC remains less well established (16). In the present case, Pro-GRP levels increased with disease progression, whereas the elevation in procalcitonin (PCT) was more pronounced and preceded the pathological confirmation of recurrence. Notably, PCT rose substantially despite the absence of fever or microbiologically documented infection and in the setting of only mild leukocytosis, raising the possibility of a noninfectious, tumor-related source of PCT elevation. Importantly, however, isolated PCT elevation should not be interpreted as evidence of tumor recurrence without appropriate assessment for infection and other causes.

If confirmed in larger studies, longitudinal PCT monitoring could potentially serve as an adjunct to conventional surveillance by identifying patients who may benefit from earlier reassessment or shortened imaging intervals Such an approach could be particularly relevant when a marked and unexplained increase occurs relative to an individual patient's prior PCT baseline. Nevertheless, there is currently insufficient evidence to define a clinically actionable PCT threshold, rate of rise, or surveillance interval for LCNEC. Moreover, treatment-related inflammation, occult infection, organ dysfunction, and other nonmalignant conditions may substantially confound PCT interpretation. Prospective thoracic oncology studies evaluating longitudinal biomarkers provide methodological precedent for incorporating serial blood-based measurements into surveillance strategies (17), but dedicated studies of PCT in LCNEC are required before such monitoring can be recommended in routine practice.

Marked reductions in peripheral T-, B-, and NK-cell counts were also observed during disease progression, suggesting substantial systemic immune impairment. Recent studies have demonstrated a heterogeneous and frequently immune-dysregulated landscape in LCNEC and other lung cancers, with impaired T- and NK-cell antitumor activity contributing to immune escape (18). However, prior chemotherapy may also alter circulating lymphocyte subsets, including B and NK cells (19). Thus, the lymphopenia observed in this patient likely reflects multifactorial effects of advanced malignancy and prior treatment and should not be interpreted as evidence against infection or as directly related to PCT elevation.

Several limitations should be acknowledged. First, this report describes a single patient with a rare histological subtype, and the observed biochemical lead time between PCT elevation and tumor recurrence cannot establish causality or generalizability. Second, molecular subtyping of the LCNEC into SCLC-like and non-small cell lung cancer (NSCLC)-like genomic subgroups was not performed. such molecular classification is increasingly recognized as biologically and potentially therapeutically relevant, and its absence limits further interpretation of the tumor phenotype. Third, conventional neuroendocrine and epithelial tumor markers, including CEA, NSE, and Pro-GRP were not measured systematically throughout follow-up, precluding direct longitudinal comparison between PCT and established serum biomarkers. Fourth, Cal was not systematically measured during the disease course, precluding assessment of a potential coordinated CT/PCT secretion phenotype. Fifth, PCT expression was not evaluated immunohistochemically in the patient's tumor tissue; therefore, a direct tumor source of circulating PCT cannot be confirmed. Finally, although extensive clinical and microbiological evaluations did not identify active infection, occult or partially treated infection cannot be excluded with absolute certainty.

Future prospective multicenter studies should incorporate serial PCT measurements together with standardized radiological response assessment, conventional tumor markers, inflammatory indices, and, when feasible, immunohistochemical or molecular characterization of tumor PCT expression. Such studies should determine whether changes in PCT correlate reproducibly with tumor burden, whether PCT elevation consistently precedes radiographic progression, and whether patient-specific kinetics provide greater diagnostic information than absolute thresholds. Ultimately, prospective validation will be required to determine whether PCT-guided surveillance can improve the timeliness of recurrence detection or influence clinically meaningful outcomes in patients with advanced LCNEC.

## Conclusion

This case describes a marked increase in serum PCT that preceded radiographic confirmation of recurrent pulmonary LCNEC by more than one month in the absence of identifiable active infection. The temporal relationship raises the hypothesis that dynamic changes in serum PCT may reflect tumor-associated biological activity in selected patients with LCNEC. However, this observation should be regarded as hypothesis-generating rather than diagnostic evidence. Serial PCT monitoring remains investigational and, if used, should complement rather than replace standard clinical assessment, microbiological evaluation, imaging, and pathological confirmation. Prospective studies are warranted to clarify the biological basis, diagnostic performance, and potential clinical utility of longitudinal PCT monitoring in LCNEC.

## Data Availability

The original contributions presented in the study are included in the article/Supplementary Material, further inquiries can be directed to the corresponding author.
